# Quantitative Assessment of Aortic Arch Conformability and Clinical Outcomes Following Thoracic Endovascular Aortic Repair

**DOI:** 10.1007/s00270-026-04481-9

**Published:** 2026-05-27

**Authors:** Sara Ffrench-Constant, Neda Oskooee, Safa Salim, Celia Riga, Richard Gibbs, Colin Bicknell, Michael Jenkins, Mohamad Hamady

**Affiliations:** 1 Department of Interventional Radiology, Imperial College Healthcare NHS Trust, London, UK; 2 Department of Vascular Surgery, Imperial College Healthcare NHS Trust, London, UK; 3 Department of Surgery and Cancer, Imperial College London, London, UK

**Keywords:** Thoracic aorta, Endograft, TEVAR, Conformability, Aorta, Aortic arch angulation, Birdbeak, Dissection, Durability

## Abstract

**Purpose:**

Thoracic stent graft conformability is believed to provide mechanical advantages during thoracic endovascular aortic repair (TEVAR); however, quantitative evidence and associated clinical outcomes remain limited. This study aimed to assess stent graft conformability across varying aortic arch configurations and pathologies, and to evaluate short- and mid-term technical and clinical outcomes.

**Materials and Methods:**

A retrospective, single-centre review was conducted of TEVAR procedures performed using the GORE cTAG stent graft over a seven-year period. Pre- and post-deployment anatomical measurements were obtained using Endosize® software to assess conformability across a range of aortic arch morphologies and aortic syndromes. Conformability was defined as a < 10% change in predefined anatomical parameters, including aortic arch angle and proximal landing zone (PLZ) angle. Demographic data, mortality, complications, incidence of bird beaking, and reintervention rates were analysed.

**Results:**

Of 189 TEVAR procedures performed during the study period, 111 met inclusion criteria and utilised the GORE cTAG stent graft. High conformability was observed following intervention, with 79% of patients demonstrating < 10% change in aortic arch angle and 88% demonstrating < 10% change in PLZ angle. Bird beaking occurred in 10.8% of patients. The overall reintervention rate was 5.4%, with greater angular changes significantly associated with reintervention (*p* = 0.039). Overall survival rates at 30 days, 1 year, and 5 years were 95%, 86%, and 80%, respectively.

**Conclusion:**

The GORE cTAG stent graft demonstrates high conformability across diverse aortic arch morphologies and pathologies, which is associated with favourable short- and mid-term technical and clinical outcomes.

**Graphical Abstract:**

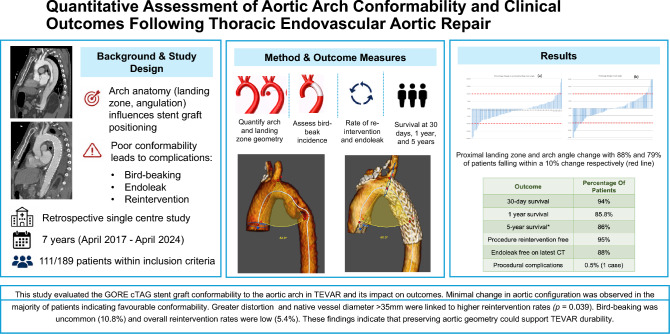

## Introduction

The short- and long-term outcomes of endografting, particularly the emergence of type 1a endoleak, are affected by a complex interplay of factors. These encompass the underlying aortic pathology, the anatomical configuration of the aortic arch—such as angulation and landing zone lengths—and technical considerations, including the mechanical properties of the graft and its post-implantation positioning or seating within the arch.

Previous studies have shown that stent conformability is likely linked to better apposition to both the outer and inner curves of the aortic arch, with less bird beaking and reduced mechanical stress on the aortic wall [1–3]. The GORE conformable thoracic aortic graft, featuring active control, was created to ‘adapt to the patient’s unique anatomy’, improve deployment accuracy, increase technical success, and enhance clinical outcomes.

Its conformability helps to minimise potential complications by promoting better contact with the aortic arch. However, there is limited research on the outcomes of more conformable stent grafts in the arch and descending thoracic aorta.

This paper aims to report on post-operative graft positioning, focusing on conformability and bird beaking of the GORE TAG Conformable Thoracic Stent Graft with Active control (GORE cTAG) in different contexts. It also explores factors that increase the risk of non-conformability and bird beaking. Additionally, the study assesses how graft positioning after surgery affects both short- and long-term outcomes, examining the links between non-conformability, bird beaking, endoleaks, reinterventions, and survival.

## Materials & Methods

### Study Design

A retrospective review was conducted at a single tertiary vascular centre, examining patients who underwent TEVAR (Thoracic Endovascular Aortic Repair) for both acute and chronic aortic presentations over seven years. Anatomical data collection was performed by two independent investigators, blind to clinical outcomes, to enhance interrater reliability. Due to the retrospective design of data collection and anonymised data processing, ethical approval was deemed unnecessary per institutional and UK Medical Research Council guidelines.

### Patient Selection

Patients were selected from a prospectively maintained registry of all endovascular devices used at the Trust. Among these, 189 patients who underwent TEVAR with the GORE c-TAG stent between April 2017 and April 2024 were identified. Patients with a non-native aortic arch (either from previous surgical repair or endovascular treatment), a proximal landing zone (PLZ) greater than 2 cm distal to the origin of the left subclavian artery, or those lacking both pre- and post-intervention imaging were excluded from the study, resulting in a total of 111 patients who met the study inclusion criteria. Similarly, although an additional 28 TEVARs were performed with other devices (82% of which were with the Medtronic Valiant graft), the scope of this paper is to analyse the outcomes of the GORE cTAG graft, and hence these patients were excluded.

### Data Collection & Analysis

**Fig. 1:**
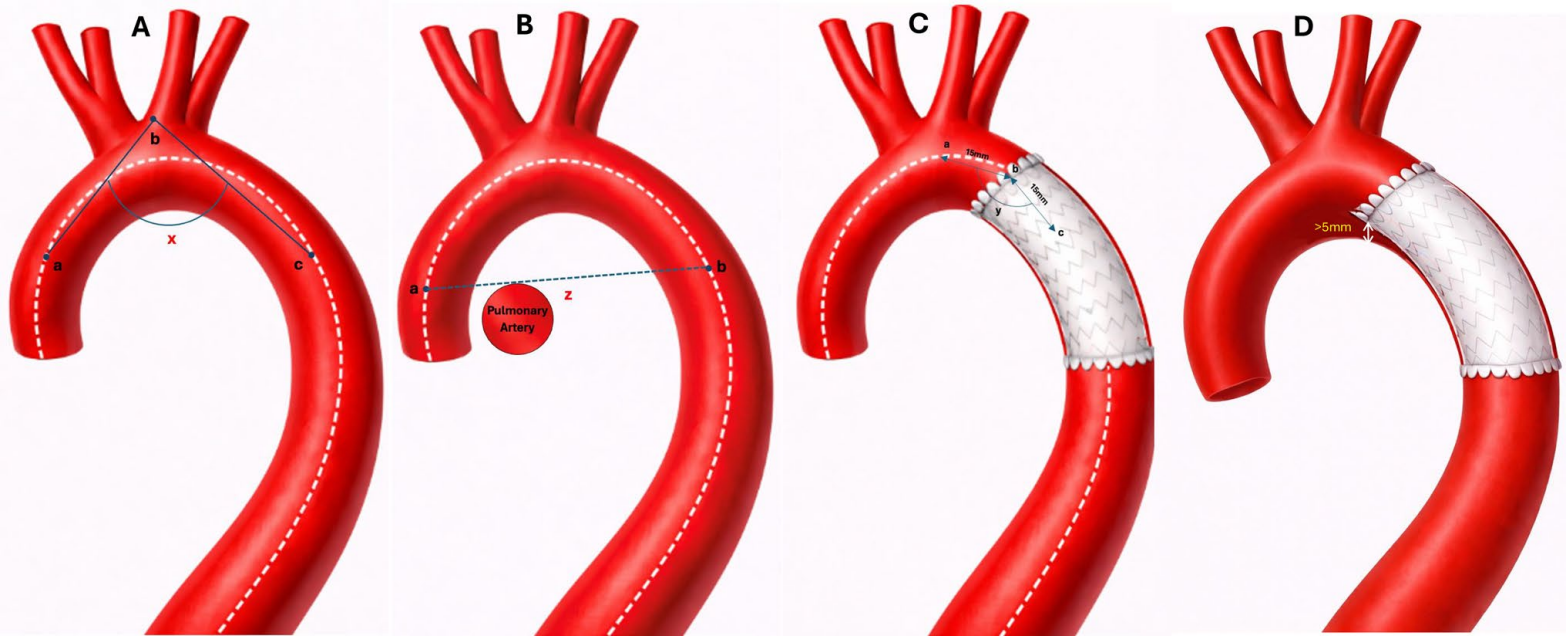
Schematic diagrams illustrating measurements of: (**A**) Aortic arch angle between (a) = mid-point between aortic valve and proximal edge of innominate artery, (b) = the highest point at the outer surface of the arch, and (c) = a point in mid thoracic aorta level with point a (**B**). Aortic arch width as the distance between a point in the mid ascending aorta (a = centreline point at the level of pulmonary trunk bifurcation) and a point in the descending aorta (b = the level of superior endplate of the T4 vertebral body) (**C**). The Proximal Landing Zone (PLZ) angle measured between two points 15 mm proximal and distal to the proximal stent cloth point. (**D**) Bird beaking defined as lack of apposition of > 5 mm between undersurface of stent cloth and inner curve of aortic arch

Arch configuration was assessed through measurement of arch angle, proximal landing zone angle and proximal landing zone diameter, on pre-operative CT and first available post-operative CT, obtained using Endosize® software. Specifically, pre-agreed upon anatomical landmarks were used to determine the following measurements:

- *Arch angle*; defined as angle between (A) mid-point of ascending aorta (between aortic valve and proximal edge of innominate artery), (B) highest point at the outer surface of the arch, (C) point in descending thoracic aorta on the centreline, level with point A in ascending aorta.
- *Proximal Landing zone angle*; measured between two points 15 mm either side of proximal stent cloth.
- *Bird-beaking assessment,* defined as a lack of apposition of > 5 mm between the undersurface of the graft and the inner curve of the arch [4]** (**Fig. 1**)**.

Not all the scans were ECG gated. Although there is a 10–20% difference in aortic diameter between systole and diastole, the lack of gating was not considered to have a significant impact on arch geometry [5].

Devices are intended to *conform* to the aorta rather than reshape it dramatically. Therefore, for the purpose of this study, bird beaking and a threshold of 10% change in geometry alterations were considered significant for lack of conformability [6]. This threshold was set a priori to identify changes likely to reflect true geometric remodelling after thoracic stent graft implantation, rather than minor variations related to image reconstruction, slice selection, or observer measurement error.

Clinical data were recorded, including demographics, underlying pathology, procedure urgency, 30-day survival, 1 year and 5-year (where applicable) survival, clinical complications and reintervention rates obtained from the electronic patient record (EPR).

Technical success was defined as accurate graft deployment without immediate, on table type 1a endoleak.

The clinical success was defined as freedom from type 1a endoleak on subsequent CT scan, sac growth, reintervention, and aneurysm-related mortality. These outcomes were selected for their quantifiable nature and relevance to longevity of the graft.

### Statistical Analysis

Categorical variables are expressed as n (%). Reintervention-free survival was analysed using the Kaplan–Meier method, with between-group comparisons performed using the log-rank test. Cox proportional hazards regression analysis was used to evaluate associations between clinical and anatomical variables and reintervention; results are reported as hazard ratios (HRs) with 95% confidence intervals (CIs). Categorical variables were compared using the Chi-square test or Fisher’s exact test, as appropriate. All tests were two-sided, and *p* < 0.05 was considered statistically significant. Statistical analyses were performed using R and Microsoft Excel.

## Results

The inclusion criteria identified 111 patients aged between 22 and 89 years, with a median age of 65. The group comprised 69% males and 31% females.

The follow-up time ranged from 10 to 93 months, with a median follow-up of 46 months.

Technical success was achieved in 100% of the studied cohort.

Median time from procedure to first post-operative CT scan was 5 days, and 79% of patients had the first post-operative CT within 30 days. The overall range was 0–352 days, with a mean of 54 days.

**Fig. 2:**
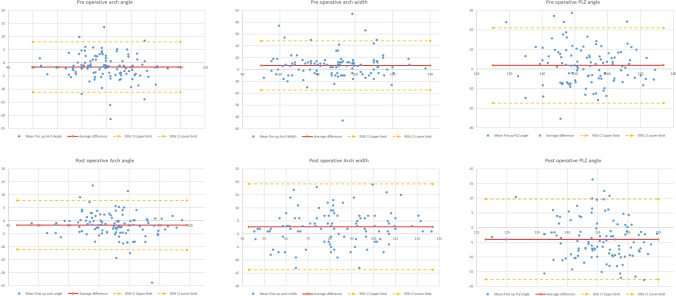
Bland–Altman plots confirming high inter-user reliability

Inter-user reliability was high, with low variation between datasets, evidenced on Bland–Altman plots in (Fig. 2).

### Underlying Pathology & Case Urgency

**Fig. 3:**
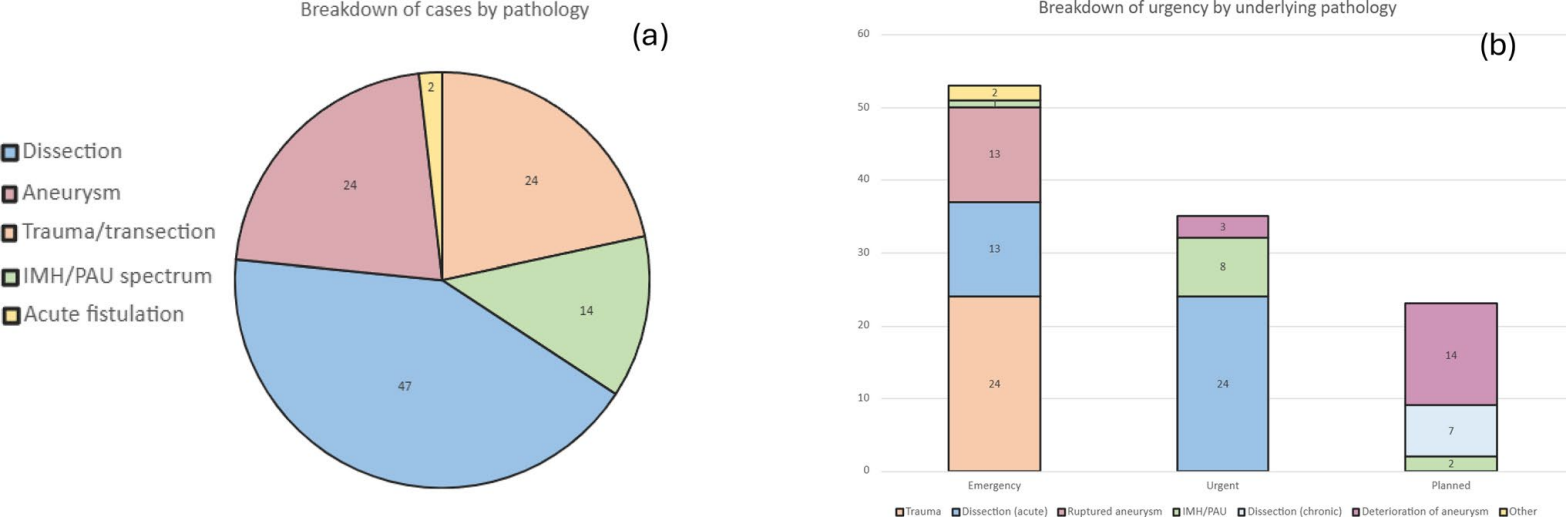
Breakdown of cases by pathology (**a**) and distribution of emergency (< 12 h), urgent (< 72 h) and planned (> 72 h) TEVAR cases with underlying clinical indication for intervention (**b**)

The most common underlying pathology was aortic dissection, representing 47 patients (42%). The majority of these (77%) involved acute or acute on chronic dissection. The remaining acute pathologies included 22% involving trauma and blunt aortic in jury (BAI), 12% fell within the IMH/PAU spectrum, and 2% were due to acute aortic conditions fistulation. Twenty-two per cent of procedures were associated with chronic aneurysmal disease (Fig. 3a).

Of the 111 TEVARs performed, 53 (48%) were emergency procedures, performed within 12 h of admission. Of these, 24 were secondary to trauma/BAI, 13 were acute dissections, 13 were aneurysmal rupture, 2 were aortic fistulae and one was an acute IMH.

Thirty-five of the TEVARs were urgent procedures (32%), performed within 72 h of presentation. The vast majority of these (24) were for acute or acute-on-chronic dissection. The remaining urgent procedures were performed for acute aortic syndrome (intramural haematoma (IMH) or penetrating aortic ulcer (PAU), (8 patients), or for rapid growth of a known aneurysm (3 patients).

In twenty-three patients were planned (20%). Fourteen of these were for enlarging thoracic aneurysms, seven were for chronic dissections, and two were for PAUs [Fig. 3b].

### Stent Graft Conformability

**Fig. 4:**
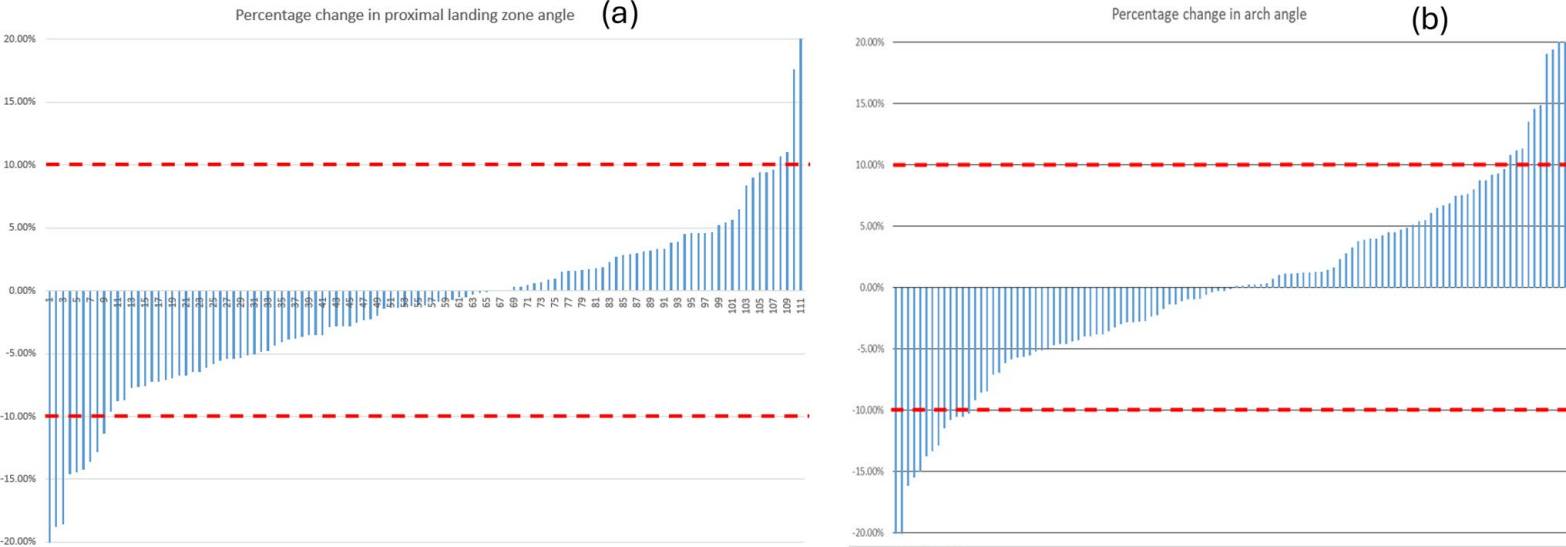
Proximal landing zone and arch angle change in all cases, showing 88 and 79% of cases, respectively, falling within a 10% change (red dotted line)

The vast majority of patients (88%) experienced < 10% change in the angle at the proximal landing zone following deployment. Similarly, 79% of patients had a < 10% overall change in the angle of the entire aortic arch (Fig. 4a and b).

Of the patients experiencing a > 10% change in proximal landing zone; 46% were related to aneurysmal pathology, while dissection and trauma represented 31 and 23%, respectively.

Conversely, of the patients with a > 10% change in arch angle; 57% were dissection, while aneurysm and traumatic represented 26% and 17%, respectively.

Although the immediate occurrence of bird beaking can be mitigated intraoperatively with the active control feature of the cTAG stent graft, this study reports a 10.8% incidence over the follow-up period.

**Fig. 5:**
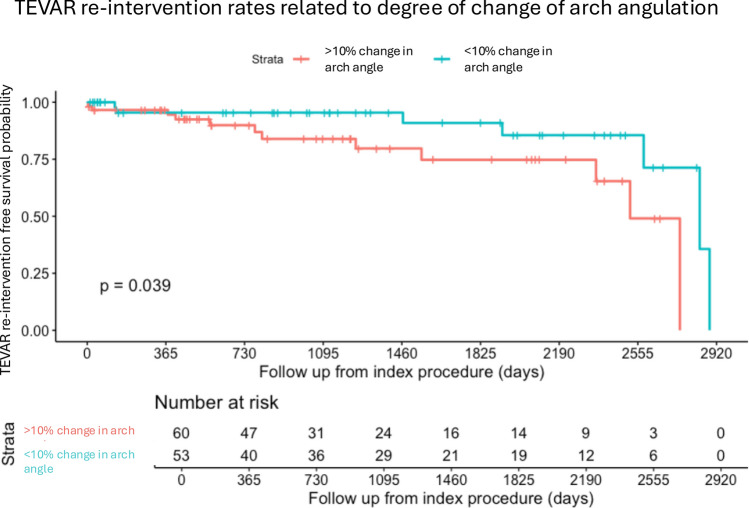
Kaplan–Meier analysis of change in arch angulation related to reintervention, demonstrating a statistically significant difference (*p* = 0.039) in reintervention rates when there is a greater change in arch angulation

Furthermore, the study demonstrated that conformability leads to a lower complication or reintervention rate, as patients who experienced a greater change in arch angle (suggesting reduced stent graft conformability) were statistically more likely to require reintervention (*p* = 0.039) (Fig. 5).

### Complications & Reintervention

Overall reinterventions rates were low, with only 5.4% of patients requiring reinterventions of the arch. All these patients required proximal extension of the TEVAR secondary to a type 1a endoleak, with one patient requiring a second proximal extension.

**Fig. 6:**
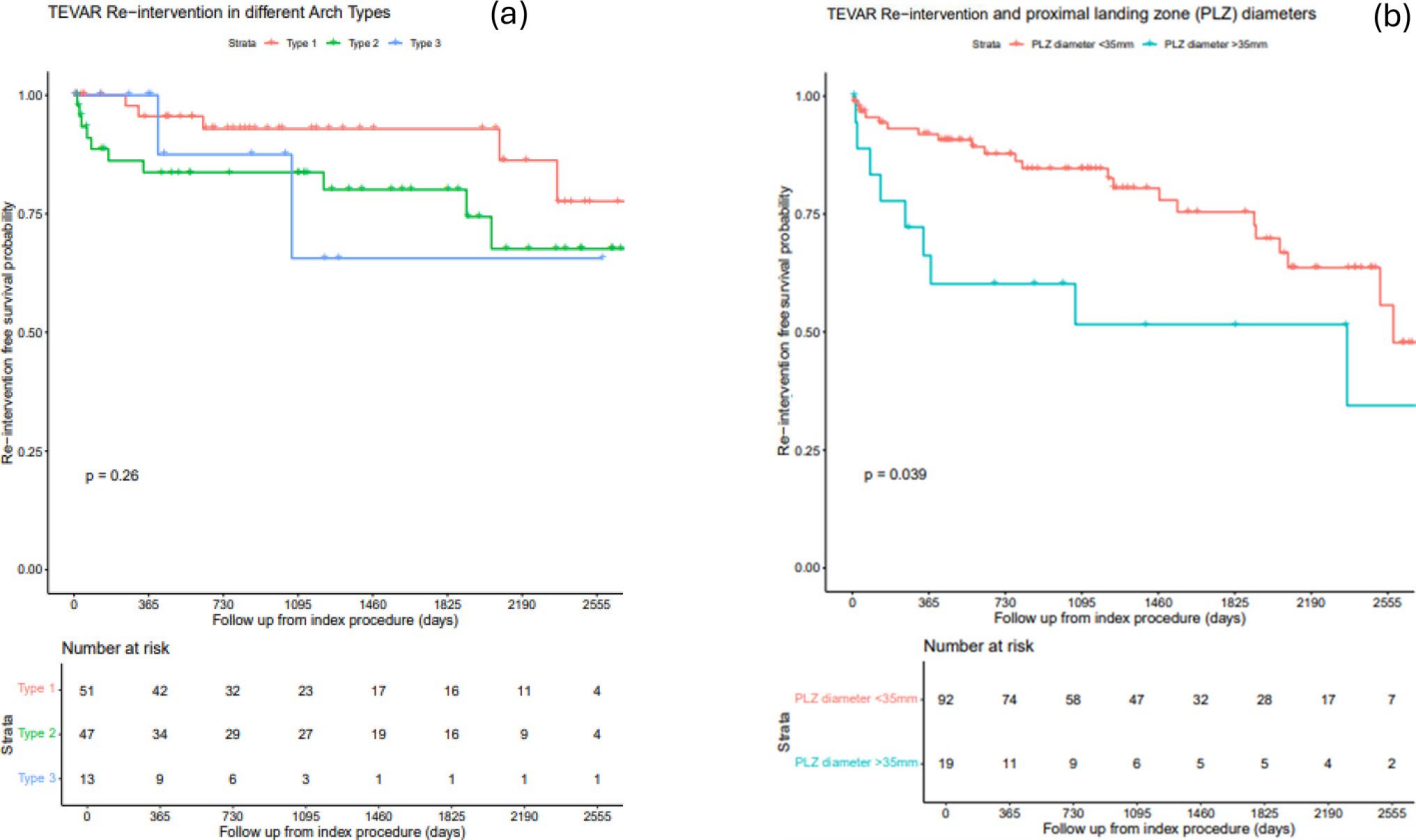
Kaplan–Meier analysis of (**a**) arch type related to reintervention, demonstrating no statistically significant difference (*p* = 0.26) between arch types and (**b**) proximal landing zone diameter related to reintervention, demonstrating a statistically significant difference (*p* = 0.039)in reintervention rates between a PLZ ≥ 35 mm and < 35 mm

There was no statistical difference between complication or reintervention rates between different arch types (*p* = 0.26), (Fig. 6a). The majority of stent deployment was in zone 3 (67%), with 28% deployed in zone 2 and 5% in zone 1, as anatomically defined by Fillinger et al. [7]. There is a statistically significant difference (*p* = 0.039) in complication rates for a PLZ diameter of > 35mm pre-intervention (Instruction For Use of 42mm) (Fig. 6b). Only in 2 procedures the device was used off IFU (PLZ diameter of 42.3mm and 45.0mm), the former of which required distal extension secondary to a type 1b endoleak but neither required proximal extension.

**Fig. 7:**
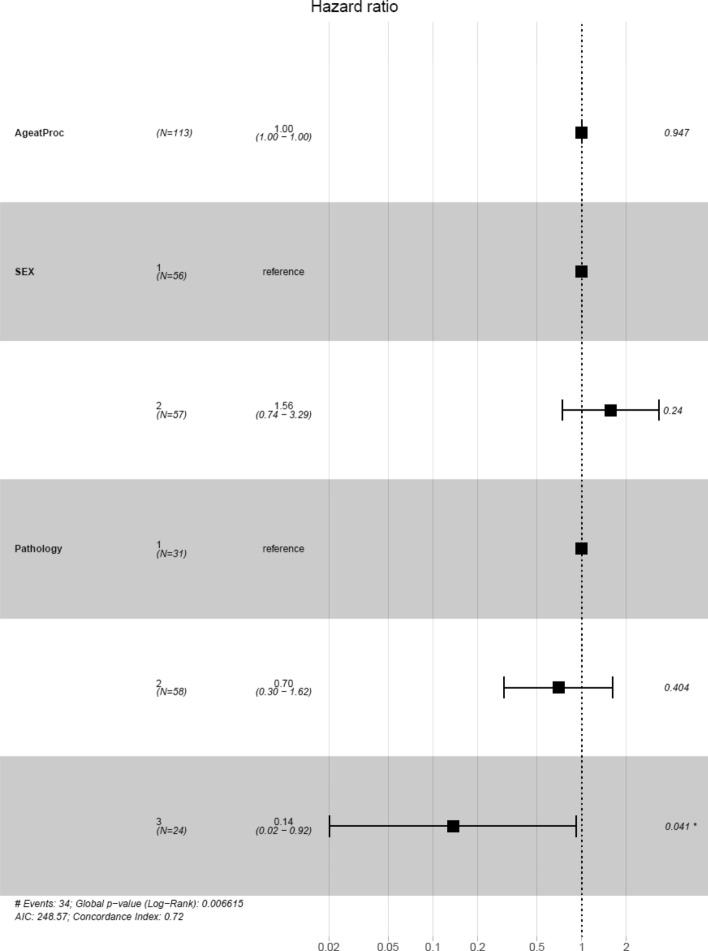
Underlying pathology of aneurysmal disease [1] is statistically more likely to require reintervention than dissection [2] (*P* = 0.4) or trauma [3] (*P* = 0.04), irrespective of gender or age. No statistical correlation between urgency of case and reintervention rates

Underlying pathology of aneurysmal disease was statistically the most likely group to require reintervention, followed by dissection (*p* = 0.4) (although this figure failed to reach statistical significance) and then trauma (*p* = 0.04), irrespective of gender or age (Fig. 7).

There is no statistical correlation between procedure urgency and reintervention rates (Fig. 7). Of the 12 patients (10.8%) with bird beaking, two underwent reintervention for type 1a endoleak.

### Survival Outcomes

Sub-set analysis according to underlying pathology was performed with respect to survival outcomes. The 30-day survival rate for chronic pathology was 100%, whilst this dropped to 92.1% for acute presentation (including dissection, IMH spectrum and trauma).

One-year survival rate for chronic pathology was 90.0% and 84.8% for acute pathology. The 5-year survival is limited by the median follow-up time of patients undergoing TEVAR > 5 years ago; the survival rate for chronic dissection patients was 71.4% (43 patients) and 88.8% in acute dissection.

## Discussion

Several studies have established the technical reliability and anatomical compatibility of the GORE cTAG endograft in patients who undergo TEVAR [8–10]. However, this study goes further by quantitatively assessing changes in the configuration of the native aortic arch pre- and post-deployment using defined anatomical landmarks across the spectrum of acute and chronic aortic pathologies, a method utilised seldom elsewhere in the literature [11], and correlating these with durability outcomes. A growing body of the literature supports the notion that poor stent apposition and stress at the graft–aorta interface may contribute to an increased risk of reintervention after TEVAR [12, 13]. This study specifically offers an objective evidence, correlating the degree of aortic arch stent-induced geometric change with complication and reintervention rates thereby suggesting a functional definition of conformability beyond target deployment accuracy alone.

Our findings demonstrate that 88% of patients had < 10% change in the proximal landing zone (PLZ) angle, and 79% a < 10% change in the overall aortic arch angle, indicating a high degree of conformability of the GORE cTAG stent to the native anatomy. Crucially, the small proportion of patients who experienced larger angular changes experienced significantly higher rates of reintervention with a type 1a endoleak representing the most common indication for reintervention. This supports our hypothesis that greater distortion of the native aortic arch following TEVAR correlates with a lower repair durability.

While current literature offers limited direct quantification of the link between stent graft conformability and clinical outcomes, existing data highlight the significance of proper device apposition and conserving native arch geometry. Böckler et al. [8] demonstrated high deployment accuracy and good arch apposition with the GORE cTAG stent graft across various challenging aortic arch anatomies. Moreover, earlier studies have stressed the need to minimise bird-beak formation to enhance procedural durability and lower device-related complications [14]. These findings collectively imply that less geometric distortion may lead to better arch apposition and, consequently, improved clinical results.

In the present study, the low incidence of bird beaking (10.8%) further supports the adaptability of the GORE cTAG to complex arch anatomy and aligns with previous reports of its conformability. Importantly, despite using a relatively low threshold for bird-beak definition (> 5 mm), only 2 of the 10 identified patients required reintervention. This observation is consistent with reports that the contemporary GORE cTAG device is associated with a lower incidence of bird-beak formation than earlier-generation devices, for which rates of approximately 44% have been described [6].

Bischoff et al. [15] also examined the early conformability of the GORE cTAG in TEVAR for type B aortic dissection, finding a high level of anatomical matching before and after the procedure, which aligns with our results. Although that study looked at geometric changes post-TEVAR, our analysis adds to this by using a reproducible measurement method with Endosize® software to quantify angular change. This approach provided a more objective evaluation of conformability and its connection to clinical outcomes.

In the current study, there was no notable difference in reintervention rates among different aortic arch types (*p* = 0.26), indicating that post-procedure results might depend more on the extent of geometric change caused by the intervention rather than the native arch classification. This aligns with the idea that changes in arch shape due to treatment—rather than the initial complexity of the arch—are more crucial for the long-term performance and durability of the device.

Bird-beak configuration after TEVAR is increasingly recognised as a clinically relevant radiological finding. Previous studies evaluating other thoracic stent graft platforms, including Captivia and Cook Zenith devices, have shown that arch anatomy influences bird-beak formation and that delayed bird beak may develop during follow-up. Bird-beak morphology and poor proximal conformity have also been implicated in device-related complications, including thoracic endograft collapse [16, 17]. These observations further underline the importance of post-implant arch geometry. However, direct evidence linking device-specific conformability to comparative long-term clinical outcomes and reintervention remains limited. As design evolution has occurred in other commercially available devices beyond Gore cTAG, further comparative studies are warranted to determine whether improved conformability is device-specific or reflects a broader advancement across contemporary endograft platforms. Moreover, although CT angiography is routinely used for post-procedural assessment, a universally standardised radiological method for evaluating bird-beak configuration and apposition has not been consistently adopted across studies.

Taken together, these findings suggest that minimising treatment-induced distortion of the aortic arch may be an important determinant of TEVAR durability. Nevertheless, these observations should be interpreted in the context of the retrospective design, potential confounding, and the absence of direct comparison with other devices. Prospective studies using standardised imaging assessment and comparative device evaluation are needed to confirm these findings.

Although aneurysmal disease accounted for the highest proportion of underlying pathology requiring reintervention (followed by dissection and trauma), the difference in the reintervention rate between aneurysmal disease and dissection did not reach statistical significance (*p* = 0.4). This challenges the suggestion that a compromised aortic wall or greater intrinsic instability alone predisposes to the need for reintervention through the increased likelihood of the aorta to redilate.

Instead, our findings support the alternative hypothesis that reintervention is more closely related to graft-induced change to the aortic configuration than to the underlying pathology itself. This view is reinforced by the lack of any significant association between underlying pathology and geometric aortic arch change following intervention; in fact, dissection accounted for a larger proportion of patients with > 10% of change in the aortic arch configuration, while aneurysm accounted for a larger proportion of the patients in which a > 10% change in PLZ was demonstrated. These findings are consistent with previous reports indicating that degenerative connective tissue disorders do not independently predict reintervention following aortic arch repair [18].

Overall survival rates in this study align well with those reported elsewhere in the literature. The overall 30-day survival rate in this study (94.5%) closely aligns with the 94.3% documented in a study conducted by Fankhauser et al. [19] and 92.5% in a study by Hu et al. [20]. Similarly, the longer-term survival rates in this study are well aligned to those elsewhere. Specifically, studies demonstrating pooled data for all-cause mortality across a range of aortic pathologies report survival rates of 73–93.7% and 43–77.7% at 1 and 5 years, respectively [21, 22], similar to the 86% and 80%, respectively, in this study.

Previous studies focused exclusively on aneurysmal disease have reported slightly lower survival rates with the study by Fankhauser et al. noting 76.4% and 52.9% survival at 1 and 5 years, respectively, and a further study by Salem et al. [23] reporting 76% and 59%. A dedicated 2008 analysis of the GORE TAG device demonstrated at 5 year survival of 68% [24]. Within our cohort, when patients with underlying aneurysmal disease were analysed separately (*n* = 17), the survival rates at 1 and 5 years were 82% and 50%, respectively, although the small sample size limits the strength of this analysis.

Although perhaps unexpected, the 5-year survival rate of acute patients was higher (88.8%) than that of chronic ones (71.4%). The likely explanation behind this is the relatively high number of young patients in this cohort, presenting with traumatic blunt injury. The 5-year survival in this cohort of patients (*n* = 24) is 100%. These patients rarely have comorbidities and, if they survive the initial trauma, are likely to have a much better survival outcome.

While the findings of this study are promising, the study is limited by the modest sample size. Future multicentre prospective studies are needed to determine whether these conformability metrics can be integrated into risk stratification models. The other limitation is the heterogeneous pathology in the studied cohort. Although disease progression can vary, potentially affecting long-term outcomes, device conformability remained consistent across the spectrum of pathology.

## Conclusion

The GORE cTAG stent graft showed favourable technical and clinical outcomes in a range of thoracic aortic pathologies and challenging anatomies. Greater post-implant change in arch angulation was associated with a higher reintervention rate, suggesting that conformability may influence longer-term outcomes. These findings are limited by the retrospective design, potential confounding, and the lack of comparison with other devices. Prospective comparative studies are required to confirm these observations.
